# Freshwater microbial metagenomes sampled across different water body characteristics, space and time in Israel

**DOI:** 10.1038/s41597-022-01749-w

**Published:** 2022-10-26

**Authors:** Ashraf Al-Ashhab, Sophi Marmen, Orna Schweitzer-Natan, Evgeni Bolotin, Hemant Patil, Diti Viner-Mozzini, Dikla Aharonovich, Ruth Hershberg, Dror Minz, Shmuel Carmeli, Eddie Cytryn, Assaf Sukenik, Daniel Sher

**Affiliations:** 1 The Dead Sea and Arava Science Center, Masada, 8698000 Israel; 2 Department of Marine Biology, Leon H. Charney School of Marine Sciences, University of Haifa, Haifa, Israel; 3 Ben Gurion University of the Negev, Eilat campus, Israel; 4 The Yigal Allon Kinneret Limnological Laboratory, Israel Oceanographic & Limnological Research Institute, P.O Box 447, Migdal, 49500 Israel; 5 Department of Genetics and Developmental Biology, the Ruth and Bruce Rappaport Faculty of Medicine, Technion–Israel Institute of Technology, Haifa, Israel; 6 Institute of Soil, Water and Environmental Sciences, Volcani Center, Agricultural Research Organization, P.O Box 15159, Rishon Lezion, 7528809 Israel; 7 Raymond and Beverly Sackler School of Chemistry and Faculty of Exact Sciences, Tel Aviv University, Ramat-Aviv, 69978 Israel

**Keywords:** Water microbiology, Metagenomics

## Abstract

Freshwater bodies are critical components of terrestrial ecosystems. The microbial communities of freshwater ecosystems are intimately linked water quality. These microbes interact with, utilize and recycle inorganic elements and organic matter. Here, we present three metagenomic sequence datasets (total of 182.9 Gbp) from different freshwater environments in Israel. The first dataset is from diverse freshwater bodies intended for different usages – a nature reserve, irrigation and aquaculture facilities, a tertiary wastewater treatment plant and a desert rainfall reservoir. The second represents a two-year time-series, collected during 2013–2014 at roughly monthly intervals, from a water reservoir connected to an aquaculture facility. The third is from several time-points during the winter and spring of 2015 in Lake Kinneret, including a bloom of the cyanobacterium Microcystis sp. These datasets are accompanied by physical, chemical, and biological measurements at each sampling point. We expect that these metagenomes will facilitate a wide range of comparative studies that seek to illuminate new aspects of freshwater microbial ecosystems and inform future water quality management approaches.

## Abstract (summary)

**Table:** 

Measurement(s)	pH measurement • temperature of water • concentration of oxygen in water • total phosphorus • ammonia • concentration of nitrate in water • concentration of nitrite in water • concentration of chlorophyll a in water • concentration of chlorophyll b in water • concentration of beta-carotene in water • concentration of fucoxanthin in water • Dinoxanthin • Diadinoxanthin • Diatoxanthin • concentration of zeaxanthin in water • concentration of lutein in water • microcystin-LR • library DNA concentration
Technology Type(s)	pH Meter • Temperature Probe Device • electron spin resonance oximetry • colorimetry • colorimetry • liquid chromatography-photodiode array detection • liquid chromatography-photodiode array detection • microcystins/Nodularins ADDA Elisa • PicoGreen Dye
Factor Type(s)	Geographic location • Isolaion source • Depth • latitude • longitude
Sample Characteristic - Environment	freshwater biome
Sample Characteristic - Location	Israel

## Background Information

**Fig. 1:**
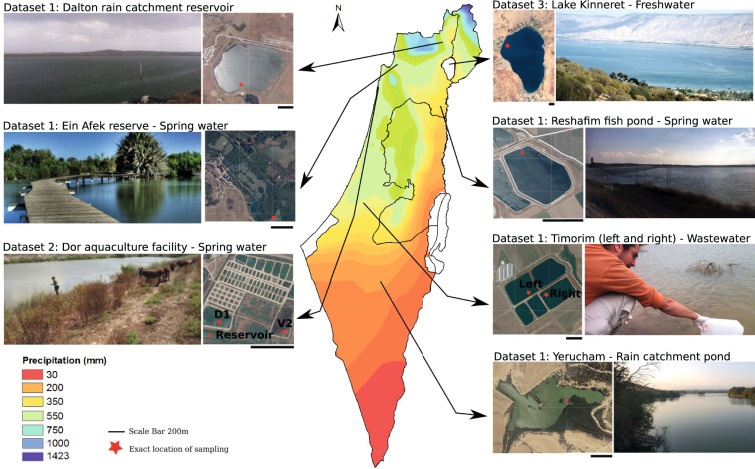
Location, satellite images (from govmap.gov.il) and photographs of the sampling sites. The color coding of the sites corresponds to rainfall gradient in (mm).

**Fig. 2:**
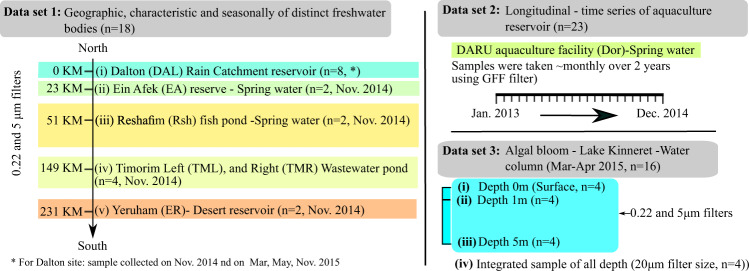
Schematic structure of the three datasets. Color coding (corresponds to rainfall gradient in (mm) similar to Fig. 1.

Microbial communities living in freshwater ecosystems (as well as brackish and marine ones) are intimately linked with water quality, and are key drivers of water biogeochemistry. Some freshwater microorganisms are potential pathogens, and others can produce toxins^1–3^. In turn, freshwater microbial communities are affected by environmental conditions such as nutrients, pH, temperature, salinity, light intensity etc., as well as by human activities^4^. Despite the importance of freshwater ecosystems, they remain relatively under-sampled across time and space^5–8^. We sampled 57 samples from different freshwater bodies in Israel (Fig. 1) to ask how aquatic microbial communities differ between different water bodies. These water bodies were selected in order to encompass several distinct characteristics of water bodies with different water uses and allocation, and were sampled over different temporal scales, resulting in three distinct datasets (Fig. 2). Dataset 1 has not been presented elsewhere, datasets 2 and 3 include metagenomics data for samples analyzed previously in the context of community structure (16 S rRNA gene amplicon sequencing^9,10^).

### Dataset 1: Differently characterized different freshwater bodies

The first dataset is from different water bodies collected during the end of summer or early fall, when water levels were relatively low (November 2014)^11–28^. The following sites were sampled (Fig. 1): (i) Dalton (DAL^11,12,15–20^), an irrigation reservoir obtaining water from several sources, including rainwater, saline springs and secondary treated wastewater. These waters are used for irrigation, and the location has a history of algal blooms that may clog the irrigation systems; (ii) Ein Afek (EA^13,14^), an aquatic nature reserve. Due to intensive use of the natural springs the water level of the reserve is managed, e.g. by receiving water from the national grid; (iii) Reshafim reservoir (Resh^21,22^), a multi-use reservoir that receives primarily spring water and is used for both irrigation and aquaculture; (iv) Timorim Left (Tim_L^25,26^), a primary wastewater pond, (v) Timorim Right (Tim_R^23,24^), a tertiary treated wastewater pond (water is then used for agriculture); (vi) Yerucham (ER^27,28^), a desert reservoir receiving both rain from its watershed and treated wastewater (Fig. 1). These sites were selected to cover multiple different characteristics and usage ponds of freshwater bodies, some of which have a history of toxic cyanobacterial blooms^29–32^. From one of these sites, DAL, we also sequenced metagenomes from March, May and November the following year, to provide a seasonal cycle (2015). At each sampling campaign, water was collected from the top 15 cm, serially filtered through 5 μm and 0.22 μm filters, representing particle-associated and free-living bacteria, respectively (more details below). In total, this dataset includes 18 metagenomes (Table 1).

**Table 1:** Summary of Dataset 1: Spatial and seasonal scale metagenome samples.

Station_ID	Sampling Dates	Number of samples	Available data categories	Latitude, longitude
Dalton	17-Nov-2014, 04-Mar-2015, 13-May-2015, 03-Nov-2015	8	Physical, Chemical (nutrients, pH, EC) and pigments	33.013770 N 35.459780 E
Ein Afek	09-Nov-2014	2		32.852979 N 35.112041 E
Reshafim	17-Nov-2014	2		32.488516 N 35.482737 E
Timmorim Lef	16-Nov-2014	2		31.716844 N 34.770119 E
Timorim Right	16-Nov-2014	2		31.715260 N 34.771648 E
Yerucham	16-Nov-2014	2		30.988393 N 34.896356 E

### Dataset 2: a two-year time series at a highly impacted reservoir

The Dor Aquaculture Research Unit is a research facility used for both aquaculture research and intensive^33–55^, semi-commercial outgrowth^9,56,57^. We followed the microbial populations from several ponds at this site over a three-year period at monthly intervals, showing that seasonality is the main driver of planktonic microbial populations in this system despite it being highly impacted by the aquaculture activity^9^. Here we present the metagenomes sequenced from 23 monthly samples over two consecutive years (2013–2014, Table 2). These samples were collected from the top 15 cm and filtered on a GF/F filter (nominal pore size 0.7 μm). A full description of the study site and the environmental conditions, as well as an analysis of 16 S rRNA gene amplicons from the same samples, is presented by Marmen *et al*.^9^.

**Table 2:** Summary of Dataset 2: Dor time series metagenomic samples.

Station_ID	Sampling Dates	Number of samples	Available data categories	Latitude, longitude
Dor reservoir	January, 2013- December, 2014	23	Physical, Chemical (nutrients, pH, EC) and pigments	32.606414 N 34.929266 E

### Dataset 3: a winter bloom in Lake Kinneret, dominated by Microcystis sp

Lake Kinneret, at the north of Israel, has been well studied for decades^58–73^, including the dynamics and causes of the winter *Microcystis* bloom^74,75^. We collected 16 samples at four time points during the winter and spring of 2015, encompassing a dynamic period in terms of environmental conditions and the dominant phytoplankton clades (Table 3). For a detailed description of these samples as well as an analysis of 16 S rRNA gene amplicons please see reference^10^. This dataset contains different sampling depth: (i) Depth-integrated samples from four dates, collected with a 20 μm vertical net (0–15 m)^58,59,66,67^; (ii) Discrete depth samples from two of the dates (0 (surface)^64,65,72,73^, 1^62,63,70,71^ and 5 meters^60,61,68,69^, two size fractions as described above).

**Table 3:** Summary of Dataset 3: Kinneret metagenome samples.

Station_ID	Sampling Dates	Depth	Filter	Number of samples	Available data categories	Latitude, longitude
Lake Kinneret	18-Jan-2015	0–15 m (integrated)	20 μm	1	Physical, Chemical (water temp, oxygen, nutrients, pH), pigments and phytoplankton counts.	(32.49692 N 35.35556 E)
	1-Mar-2015	0–15 m (integrated)	20 μm	1		
		0 m (surface)	0.22 and 5 μm	2		
		1 m	0.22 and 5 μm	2		
		5 m	0.22 and 5 μm	2		
	22-Mar-2015	0–15 m (integrated)	20 μm	1		
	15-Apr-2015	0–15 m (integrated)	20 μm	1		
		0 m (surface)	0.22 and 5 μm	2		
		1 m	0.22 and 5 μm	2		
		5 m	0.22 and 5 μm	2		

### Metadata

These metagenomic datasets are accompanied by various environmental measurements. The methods are briefly presented here, with more details available in^9^. Briefly, electrical conductivity (EC), dissolved oxygen, temperature and pH were measured *in-situ* using field probes (Eutech instruments, Singapore). Ammonia, total phosphorus, NO_2_, NO_3_ and PO_4_ were measured using a AA3 Segmented Flow Multi-Chemistry Analyzer (SEAL Analytical, Germany), following the manufacturer protocols (Ammonia: Method no. G-327-05 Rev. 7 - Fluorescent method; Nitrate and Nitrite: Method no. G-172-96 Rev. 17 and Phosphate: Method no. G-297-03 Rev. 5). Toxin concentrations were measured using the Microcystins/Nodularins (ADDA) Elisa from Abraxis, following the manufacturer protocol.

Photosynthetic pigments were measured using ultra-performance liquid chromatography (UPLC), using a method adapted from the LOV protocol^76^. Briefly, pigments were extracted for 3 hours in absolute methanol in the dark, the filtered through 0.2 µm PTFE membranes (Pall Life Sciences, New York, NY, USA), and preheated to 30 °C. 10 µl were injected into an ACQUITY UPLC system (Waters Corporation, Milford, MA, USA) equipped with photodiode array detector. Separation was performed on a C8 column (ACQUITY UPLC BEH, 50 mm column length) using a linear gradient (solvent A - 70:30 methanol: 0.5 M ammonium acetate; Solvent B - 100% methanol). Peaks were identified based on their retention time and absorbance spectra, and quantified by comparison to standards of Chlorophyll a, Chlorophyll b, Chlorophyll c_2_, Zeaxanthin, β-carotene, Diatoxanthin, Dinoxanthin, Fucoxanthin and Peridinin (DHI Laboratory, Hørsholm Denmark).

Extracted DNA and archived samples are available for further research by the community (please contact the corresponding authors). The complete dataset contains ~416.5 gigabases of raw sequence data (Supplementary Table 2). Dataset 1, 2 and 3, provides opportunities to compare and contrast variations within and between these different freshwater sites across temporal, seasonal, spatial, water quality and environmental conditions.

## Methods

Water samples for dataset 1 were collected from surface water (top 15 cm) using a hand-held bucket, filtered in the field using hand-held vacuum filters, and were partitioned into two size-fractions by sequentially filtering the water onto a 5 μm filter (Millipore) and 0.22 μm filter (Supor-200 Membrane Disc Filters, 25 mm; Pall Corporation, East Hills, NY). Samples were frozen on dry ice in the field. For dataset 2, samples were collected using a hand-held bucket from surface water (top 15 cm), transferred to the lab in the dark (30 minutes away) and filtered onto GF/F filters (Whatman glass fiber, 25 mm nominal pore size 0.7 μm). For dataset 3, samples were collected first as integrated samples; by a vertical phytoplankton net (20 μm mesh, 0–15 meters) followed by filtration on 5 μm filters. Additional water samples from three discrete depths - 0 (surface), 1, and 5 meters – using Niskin bottles. For all samples, the filters were preserved in a 1 ml of storage buffer (40 mM EDTA, 50 mM Tris-HCl, 0.75 M sucrose) and stored at −80 °C until DNA extraction.

DNA extraction was performed using a semi-automatic extraction method including both Chemical and Mechanical extraction as follows; (i) lysis buffer (20 mM Tris⋅Cl, pH 8.0, 2 mM sodium EDTA, 1.2% Triton® X-100) (DNeasy Blood & Tissue Kit, Qiagen) with 30 μL of lysozyme were added to the samples and incubated for 30 min at 37 °C. 25 μL or proteinase K and 200 μL of buffer AL from the kit were added and the tubes were incubated at 56 °C for 1 h with agitation. Chemical lysis was followed by mechanical lysis using 3 mm stainless steel beads (30 Hz for 1.5 min, TissueLyser LT, Qiagen). Finally, DNA was extracted using a QiaCube robot following manufacturer protocol. For metagenomic library preparation, samples were sheared using Nextera XT library preparation kit with the default library linkers and adaptors. Then, libraries were quantified and their quality verified using the AATI Fragment analyzer for a target library size of ~420 bp, including adapters (see Supplementary Table 2 for library concentrations and intensity). The libraries were sequenced on an Illumina NextSeq-500 platform producing 150 bp paired-end reads at DNA Services Facility of the University of Illinois (Chicago, USA) to allow sequence assembly of 300 bp with minimum overlap of 8 bp. The DNA concentration, quality and obtained number of sequences for each library are reported in data file 5.

## Data Record

The raw, unprocessed Illumina sequencing reads (fastq files) for all metagenomes are available from the NCBI Sequence Read Archive (PRJNA497963 and PRJNA488159). All sample accession numbers^11–28,33–55,58–73^, sample date/location, and library concentration and intensity for each metagenome can be found in Supplementary Table 2. The first^11–28^ and third^58–73^ datasets (Tables 1 and 3) were deposited under NCBI project number PRJNA497963, Supplementary Table 2). The second^33–55^ dataset (Table 2) was deposited under NCBI project number PRJNA488159 (Supplementary Table 2).

All various environmental measurements (EC, oxygen, temperature, Ammonia, total phosphorus, NO2, NO3, pH, photosynthetic pigments and toxin concentrations) (Supplementary Table 1) and samples metadata file (Supplementary Table 2). We note that not all data are available for every sample. Data pertinent to a specific dataset (e.g. *Microcystis* concentrations in dataset 2) are marked as “-“ in Supplementary Table 2 when not collected. Other missing data are marked as “NA”.

## Technical Validation

Total number of bases (Kbp), GC %, File size (Kbytes), initial and retained Sequences number for all datasets are presented in Supplementary Table 1. For the first data set (multiple water bodies^11–28^), the 18 samples were multiplexed and sequenced in one run resulting in a median of 404.1 million raw paired-end reads for each sample (range: ~16.2–24.5 M reads). For the second dataset (DARU time series^33–55^), 23 metagenomes were multiplexed and sequenced in one run resulting in a median of 762.4 million raw paired-end reads per sample (range: ~0.4–51.1 million). For the third dataset (Kinneret cyanobacterial bloom^58–73^) 16 metagenomes were multiplexed and sequenced in one run resulting in a median of 239.0 million raw paired-end reads per sample (range: ~13.1–16.8). The current study did not include any spike-in DNA or a negative control. However, multiple negative controls were performed on the DNA during the amplicon sequencing described in^9^, and no cross-contamination was observed.

## Supplementary information

Supplementary Table 1

Supplementary Table 2

## Data Availability

Not applicable, we submitted to the original sequences, therefore no code or software was used.
